# University Virtual Learning in Covid Times

**DOI:** 10.1007/s10758-021-09533-2

**Published:** 2021-07-20

**Authors:** Verónica Marín-Díaz, Eloísa Reche, Javier Martín

**Affiliations:** University of Cordoba, Córdoba, Spain

**Keywords:** Training platform, Learning, Virtual teaching, University student, Moodle

## Abstract

Online training is demanded in the ubiquitous society we live in, and this is especially true if we consider the current situation at universities due to the Government issuing a state of alarm decree which requests all citizens to remain at home. The goal of this study is to determine the opinion of university students from different Spanish campuses on e-learning platforms, by the means of a descriptive and correlational study design, with N = 431. The results reveal that there is still a long road ahead to ensure that these tools work optimally to enable professors to fully exert their teaching profession. We can conclude that the online teaching system needs to be improved regarding the technical service that the university offers.

## Introduction

Non-classroom learning has been evolving in recent decades as a result of technological developments, which have evolved almost on a daily basis. It is indisputable that, at a higher level, the use of teletraining platforms (hereinafter PTM) have carved a niche for themselves. As of today, and without a doubt, modalities such as ubiquitous learning have underlined the fact that the teaching and learning process have become increasingly open and flexible. Papers written by experts such as the Cole et al. (2014) and Marín-Díaz et al. (2016), have discussed that elements such as satisfaction with the platform, the ability to interact with the content, availability, technology, not only connecting to the virtual campus but, also interacting with the materials which are supported in this learning format, as well as the skills of teachers to "work", are the main elements needed for the Online training platforms to succeed.

**Fig. 1:**
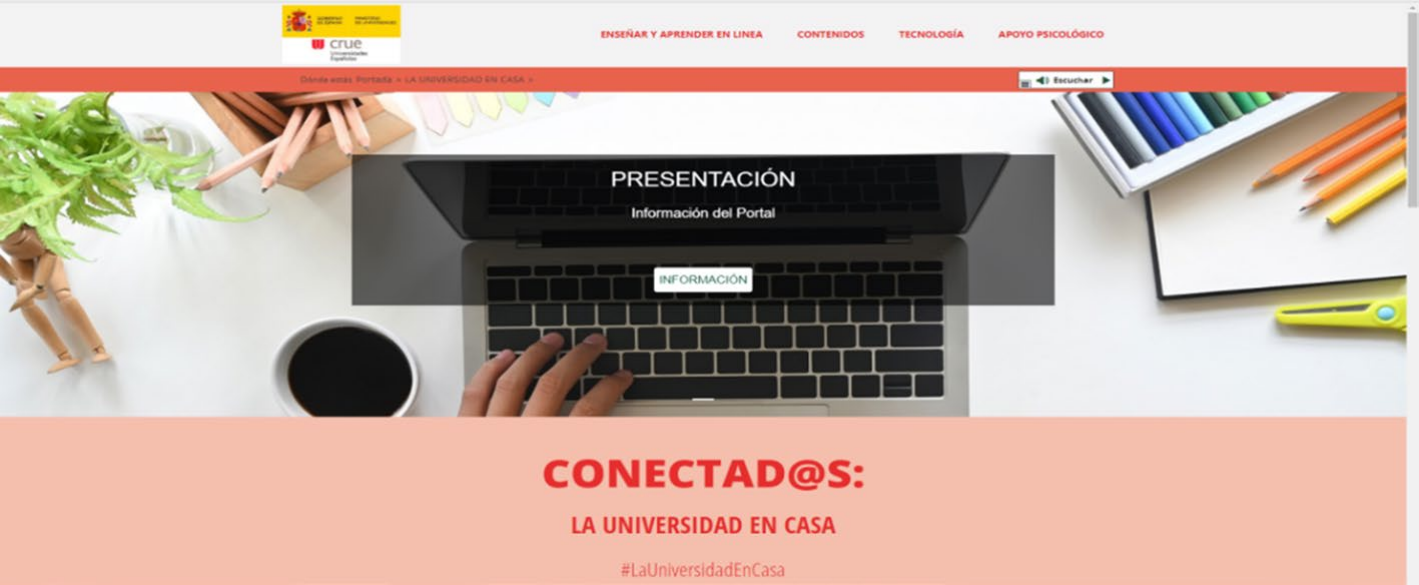
Plataforma Conect@s

In this sense, now that citizens are confined to their homes, it has become indisputable that virtual learning is the key to training processes that do not stop. In the case of Spanish higher education, after a few days of saturation of their own virtual campuses, the Ministry of Universities, together with the Conference of Rectors of Spanish Universities (hereinafter CRUE) created, under the supervision of the two public universities operating online in Spain, the National Distance Education University (hereinafter UNED) in Madrid and the Open University of Catalonia (hereinafter UOC) in Barcelona, a portal called "*CONECTA @ s: The home university*", aiming to help teachers adapt the materials which had been initially designed for face-to-face teaching, with elements such as tutorials for the creation and presentation of materials, video conferencing, evaluations, etc (Fig. 1).

Firstly, as teachers, we should be thankful for the initiative. Nonetheless, we have observed that a key element of this process was not taken into account: the student, given that said resource was designed with the teacher in mind.

Discussions in forums and chats employed in subjects taught online at university are full of expressions of gratitude from the students, who are most grateful for the efforts by the teachers to try to maintain a certain level of education, yet they have also reflected, in those small informal conversations, that the system has been saturated with the use of tools that they did not use before this situation and that, in some cases, they did not consider relevant. In some other cases, tools were used which students did not even know how to use (Maz et al., 2012; AUTHOR). This shows that the results from some research studies which identified the crucial elements for virtual campus to succeed (greater interaction of students with the tools available on the platform) (Binyamin et al., 2019; Gilmore, 2020; Gómez & Moreno, 2018; Ohliati & Abbas, 2019; Villalón et al., 2019), had not been fully developed.

For this reason, the questionnaire created by Maldonado in 2012 was utilized as the starting instrument, and was applied to a sample of students from a university in the south of Spain. The main result obtained was that female students obtained the greatest value and possibilities from the PTM. On the other hand, the scarce link teachers felt with the use of platforms as a means to convey their teaching strategy also became evident.

## State of the art

We agree with Puerta-Cortés and Carbonell (2013) that in most cases, Information and Communication Technologies (ICT) can contribute with the improvement of the quality of life of people, because they allow us to interact differently with society, fostering a greater diversity from social, economic, interpersonal, and educational points of view. On the other hand, we agree with the approach by Macgilchrist, Allert and Bruch (2020), who indicate that today, the future of education is drawn in three scenarios (*smooth and competent users; digital nomads, and collective agency*), and that society in general, and educational environments in particular, should think about which models must be followed. However, the situation the world is currently experiencing, in our view, has demonstrated that we are heading towards the third scenario pointed out by these authors, where solidarity and virtual learning become the cornerstone of the training process, in favour of reducing the digital educational gap, which occurs at times when all teaching, as it is presently occurring, becomes virtual (Almoguera, 2020).

Thus, online training is now a reality that brings benefits and drawbacks to its users, -teachers and students-, which implies a redefinition of its methodological perspective. In some cases, teachers and students must abandon both of their traditional teaching and learning strategies, which are based on the master class (lecture) and the face-to-face class (Villalón et al., 2019). Moreover, in recent decades, as sponsored by Europe, mainly due to the implementation of the European Higher Education Area (hereinafter EHEA), teachers have had to incorporate new ways of introducing the contents. Therefore, in teacher’s forums, it is now common to find innovation proposals framed, for example, within problem-based learning supported by digital resources and developed through online training platforms (Tejada & Thayer, 2019), or within the so-called equally digitized service learning (Mdutshekelwa & Mostert, 2019).

With regard to the university students, some authors such as Pérez-Berenguer and García-Molina (2016) have defined them as digital students who can find face-to-face learning tedious, despite the fact that the key to their learning in current technological society is based on the type of relationship that they have with the e-learning systems. Thus, it must be borne in mind that this relationship can be two-sided. On the one hand that the students have already had previous contact with this type of telematic systems and therefore we talk about digital students, and on the other hand, this is biased, since they may only know some tools that the PTM could provide or they may face something entirely new (Gómez & Moreno, 2018), which could result in situations that generate stress or rejection. In both cases, it is their attitude towards them which will determine their connection with the learning process, at the same time that it will determine their relationship both with their classmates and teachers and in general, with the subject studied (Sonmez & Koc, 2018).

In any case, PTM have been described as dynamic elements that promote innovative teaching and constant growth, and a way to promote a rich and varied learning (Sonmez & Koc, 2018). Their presence in university campuses has already become widespread, unlike its employment by all teachers. The reasons for this presence are several and range from economic aspects, -as is the case of universities in Andalusia, where the the Regional Government has a contract with higher education institutions that stipulates the use of PTM for teaching. This contract is then utilized as an economic indicator, with a greater presence of virtualized subjects resulting in a greater overall score for the institution, leading to a budget increase-, to education aspects, as shown by the growth of the online training offerings from different agencies, companies, universities, etc., or from business, where the versatility of these formats is highly valued.

Moreover, the use of PTMs implies that users (teachers and students) interact and cooperate in promoting simultaneous learning. This is also collaborative, aside from cooperative, as it tries to overcome the initial limitation of physical distance. For this reason, the pedagogical knowledge, together with the technological knowledge of the platform become cornerstones of its success as a training tool. If we focus on the case of the PTM *Moodle*, we found that in general, this platform offered a number of communication tools as well as different work and evaluation tools, which have been implemented over the years.

In its beginnings, like any resource, it had a series of limitations for both teachers and students. The tools for the creation of evaluation questionnaires, the use of discussion forums or the uploading of video files, and the incorrect reception of messages (Maz et al., 2012; Felpeto-Guerrero et al., 2015) were outlined as conflicting. Today, the difficulties revolve more around the Internet connection, the place where we can connect, and the quality of the materials uploaded by teachers, together with the quality of the content of the debates generated by the students, the organization of group work or online tutoring (AUTHOR), to name the most relevant elements.

Studies conducted by Mpungose (2020) with first-year South African university students highlighted the difficulties experienced by the students when using Moodle in their learning process, mainly due to their lack of knowledge about this platform. However, the students from the Faculty of Medicine, who participated in the teaching innovation program developed at the University of Havana (Cuba), indicated that they considered this new way of evaluating their learning progress as very valuable, mainly due to the versatility of the platform. Nevertheless, the study showed that the main weakness found was due the lack of knowledge of the professors about it (Pomares et al., 2020). We believe that these diverging results demands a detailed study of the relationship between the platforms in general, and Moodle in particular, with the students and the professors as well.

On the other hand, we cannot forget that the bi-directionality offered by *Moodle* to the teaching–learning process is its most valuable feature in our opinion (AUTHOR). In this way, the availability of content and its accessibility at any time and place, which save both time and money, are variables that make *Moodle* a platform with a high educational value in the area of Educational Sciences (Rivadulla-López, 2015). Along this line, we find the works by Hernández-Amoros et al. (2020), and Romero-Díaz de la Guardia et al. (2021). These works show that its use in this area is positively evaluated by all the participants in the higher learning process. As a result, as we have already pointed out, the study of this “marriage” acquires a greater importance at the present time, when online teaching support provided by many different platforms is on the rise. Thus, as some of the main points of this study, we find the connectivity problems with the platform (AUTOR), which platforms are utilized (Setiawan, 2021), or the performance and effort expectations, the social influence, and the social isolation of the students, and their relationship with the Platform (Raza et al., 2020).

## Method

This research was carried out using a quantitative paradigm and an ex post facto study, using a descriptive and correlational design (Mateo, 2012). The goal was to determine the opinion of college students from a Spanish university in general, and from Andalusia in particular, on the learning platforms offered by the universities. For this, the following specific objectives were developed:

1. To determine which are the most utilized tools of the PTM during their learning process.
2. To evaluate the PTM interface used by the university.
3. To assess the figure of the teacher against their learning process mediated by the PTM.
4. To determine the computer service (classroom and connection) that provides technical support to the PTM offered by the university.
5. The students of the Primary School Education Degree are more satisfied with the training through Moodle.

The working hypotheses generated as a result of these objectives are:

- **Hypothesis A** Women are more favorable to online training.
- **Hypothesis B** Younger students have a better assessment and use of PTM.
- **Hypothesis C** Primary School Education degree students are more satisfied with the training through Moodle.

### Sample

**Fig. 2:**
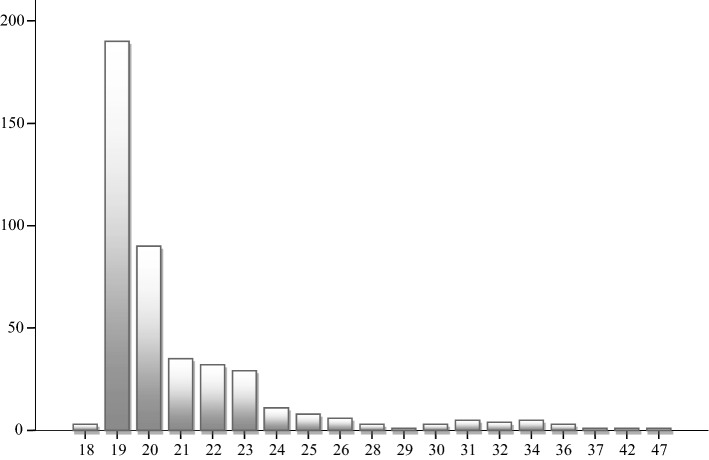
Distribution of the sample, according to age

The population under study were university students in the area of Social Sciences, Education branch, from the University of Córdoba (Spain). The type of sampling was incidental (Mantilla, 2015), with N = 431, where 35.5% were women and 64.4% were men. Regarding their age, we verified that the population group was 21.07 years-old on average (SD: 3.651) (see Figure 2), with the following distribution according to their degree: 83.8% studied Early Childhood Education, and 16.2% were enrolled in the Primary School Education Degree.

As for the gender + degree combination, it was found that 90.84% of the men were enrolled in the Early Childhood Education Undergraduate Degree, while 9.16% were enrolled in the Primary School Education degree, and in the case of women, 79.9% were enrolled in the Early Childhood Education Degree, and 20.1% in the Primary School Education Degree.

To discover their possible knowledge related to the use of platforms for online learning, they were asked if they had completed any training course in an online format, finding that 91.2% had not carried out any type of training activity, while 8.8% mentioned having done so.

### Instrument

To carry out this research, the instrument created by Maldonado in 2012 and validated by AUTHOR was utilized. The instrument was initially comprised of 76 items, with a 5-option Likert-type response scale (Matas, 2018).

In order to check if the instrument continued to maintain the reliability and validity results as the original, various statistical procedures were applied. Regarding reliability, the Cronbach’s Alpha test was performed, with a value of 0.903 obtained. This point to the stability of the items according to Mateo (2012); thus, the data from the original are maintained (Maldonado, 2012).

Regarding the construct validity of the instrument, an exploratory factor analysis (EFA) was performed, -following the parameters from García et al. (2000) and Grant and Fabrigar (2011)-, which signalled to the existence of 6 factors, just as the original. However, this analysis also eliminated 20 items that obtained scores below 0.30, as according to López-Roldán and Fachelli (2016), they lacked significance.

In the EFA performed, the Pearson’s correlation matrix was utilized, along with the procedure "Optimal implementation of parallel analysis" (Timmerman & Lorenzo-Seva, 2011) for determining the number of factors, and the «Maximum Likelihood» extraction method for common factors with a «Weighted Oblimin» rotation criteria (López-Roldán & Fachelli, 2016), using the SPSS 23 statistical package. A correlation value of 0.000 was obtained, with a KMO of 0.730, and a Bartlett’s sphericity test result of *p* = 0.000. The factors extracted explained 60.813% of the total variance and the matrix of the rotated components allowed us to observe that the factorial weight of each of the items had loads higher than 0.30, considering the parameters by Morales (2011), with the results obtained shown in Table 1.

**Table 1:** EFA rotated factors matrix

Item	Factors					
	1	2	3	4	5	6
1. The organizational structure of the subject is clearly shown within the PTM: competences	.799					
2. The organizational structure of the subject is clearly shown within the PTM: objectives	.795					
3. The organizational structure of the subject is clearly shown within the PTM: contents	.793					
4. The organizational structure of the subject is clearly shown within the PTM: Methodology	.787					
5. The organizational structure of the subject is clearly shown within the PTM: assessment	.744					
6. The organizational structure of the subject is easily accessed within the PTM	.707					
7. In the PTM it is easy to identify where to consult the program of the subject	.578					
8. The PTM allows learning from anywhere		.803				
9. I consider tele-training necessary due to the lack of training offer in my environment		.781				
10. The PTM allows learning at any time		.757				
10. The activities that are managed through the PTM encourage me to get involved in my learning		.700				
12. PTM promotes the ability to investigate		.689				
13. The PTM is a tool with which I learn more dynamically		.684				
14.The PTM makes my learning more flexible		.631				
15. The PTM facilitates active learning		.627				
16. The PTM is a tool with which I learn more easily		.562				
17. The PTM makes the class session more relaxed		.545				
18. I prefer that problems arise and work on them without a fixed content scheme through the PTM		.505				
19. The PTM means greater autonomy in the learning process		.493				
20. I prefer to learn in organizational subjects on topics sequenced through the PTM		.500				
21. To learn in teletraining I prefer to take short courses (no more than three months)		− .766				
22. I prefer to participate in blended subjects		− .585				
23. The PTM interface is dynamic			.775			
24. It is easy to carry out individual and collaborative activities through the PTM			.717			
25. The PTM interface is didactic			.713			
26. PTM interface is motivating			.570			
27. The PTM is easy to use since its navigation is friendly			.559			
28. It is easy to know my progress and my evaluations through the PTM			.517			
29. PTM accessibility is fast			.487			
30. Teachers provide me with a quick answer to my questions through the PTM				.817		
31. Teachers provide me with an adequate answer to my questions through the PTM				.792		
32. The teachers, through the PTM, suggest additional strategies so that I can carry out my activities in a better way				.609		
33. I consider it important that teachers take time to guide me on the subject in the same PTM				.526		
34. I manage the chat to communicate with my friends					.888	
35. I manage the chat to communicate with my university colleagues					.872	
36. I manage the forums to communicate with my friends					.810	
37. I manage email to communicate with my friends					.783	
38. I manage the forums to communicate with my university colleagues					.779	
39. I manage email to communicate with my college classmates					.765	
40. I use RSS to stimulate different spaces on the Internet (blogs, wikis, web pages, etc.)					.716	
41. I use blogs for personal activities					.716	
42. I use podcasting for personal activities					.683	
43. I use wikis for academic activities					.680	
44. I use wikis for personal activity					.676	
45. The PTM allows me to interact with people from other educational centers					.544	
46. I use blogs for academic activities					.493	
47. I manage the forums to communicate with the teaching staff					.462	
48. I manage the chat to communicate with the teaching staff					.441	
49. I manage email to communicate with teachers					.417	
50. I use podcasting for academic activities					− .419	
51. The computers in the computer room are well equipped						.782
52. The computer room has enough computers						.758
53. I connect to the subjects present at the PTM in my home						.727
54. The computer room schedule gives me access to computers						.725
55. I connect to the subjects present in the teletraining platform (PTM) in the faculty						.711
56. Wi-Fi is easily accessible from any area of the university						.616

In order to check the reliability of the items resulting from the EFA, Cronbach’s Alpha was performed again based on the 6 established dimensions, as observed in Table 2. We verified that two of the dimensions had a high reliability, three were moderate, and one low.

**Table 2:** Reliability of Dimensions

Dimensions	Alpha
1. Structure of the PTM (7 items)	. 907
2. Perceived utility of the PTM (15 items)	.781
3. PTM interface (7 items)	.833
4. Visions l to work the faculty in the PTM (4 items)	.547
5. Uses of the tools (17 items)	.788
6. Hours and place of usage/connection (6 items)	.618

## Results

The analyses conducted in this work, through which we tried to provide an answer to the objectives described, were the descriptive analysis through the measurements of central tendency and dispersion (mean and standard deviation, M and SD). These were used to show the opinions of the students associated to their perception on the Moodle platform, through the use of the 6 dimensions found after the EFA, hypothesis testing with the Student’ t and Cohen’s D tests, in the case of dichotomous variables, and an ANOVA in the case of polytomous ones, to verify the existence of differences between the dimensions of the questionnaire, and the variables gender, age, and degree. Lastly, a correlational study was conducted with the use of Pearson’s correlation to elucidate the relationships between the 6 dimensions.

Dimension 1: Structure of the PTM.

Regarding the organization of the PTM in terms of its clarity (item 1, M = 3.86; SD = 0.740), the objectives (item 2, M = 3.91; SD = 0.658), the contents (item 3, M = 3.95; SD = 0.658), the methodology (item 4, M = 3.82; SD = 0.703) and the evaluation (item 5, M = 3.86; SD = 0.739), as well as the easy possibility of accessing the organizational structure of the subject (item 6, M = 3.98; SD = 0.703), we verified that the participants had a quite positive attitude, beyond simple indifference. Also, they positively evaluated the ease with which the program of the course could be found (item 7, M = 4.06, SD = 1.1207).

## Dimension 2: Perceived utility of the PTM

It is striking that the participating students considered that the PTM allowed learning at any time (item 10, M = 4.01; SD = 0.752), at the same time that they disagreed with participating in blended learning-based courses (item 22, M = 2.61; SD = 1.164). Just as dimension 1, they were indifferent to the rest of the items.

## Dimension 3; PTM interface

With respect to this dimension, the students were also indifferent in their assessment, with the set of items obtaining a mean of M = 3.62 and a SD = 0.843.

## Dimension 4: Points of view about the work of teachers in the PTM

Just as in the dimensions above, the students were indifferent to how the teacher performed his or her work in the PTM, although they considered it important that teachers devote time to guide them around the course in the PTM (item 33, M = 4.00; SD = 0.694).

## Dimension 5: Use of tools

Regarding the student’s use of the tools provided in the PTM, it was observed that they were in agreement as to using the chat to communicate both with their friends (item 34; M = 4.07; SD = 1.240), as with their university classmates (item 35; M = 4.03; SD = 1.207), and email to communicate with their professors (item 49, M = 4.49; SD = 0.661). On the other hand, they were not in agreement with the following statements: management of forums and email to communicate with their friends (item 36, M = 2.29; SD = 1.049; item 37, M = 2.81; SD = 1.248), management of the forums to communicate with their university classmates (item 38, M = 2.70; SD = 1.127), and the chat with teachers (item 48, M = 2.95; SD = 1.212); In addition, they also disagreed on the use of blogs, podcasting, wikis for their personal activities (item 41, M = 2.91; SD = 1.146; item 42, M = 2.56; SD = 1.003; item 44, M = 2.89; SD = 1.072) and podcasting in their academic activities (item 50; M = 2.65; SD = 0.981). Finally, it is significant that they did not consider that the PTM (in this case Moodle) allowed them to interact with people from other educational centers (item 45; M = 2.77; SD = 0.952).

## Dimension 6: Hours and place of use / connection

In this last dimension, the students declared to be in agreement with the statements from items 53 (I connect to the subjects present in the PTM at home; M = 4.16; SD = 0.832), and 55 (I connect to the current subjects in the training platform [PTM] at the faculty; M = 4.08, SD = 0.885), and disagree with item 56 (It is easy to access the Wi-Fi from any area of the university; M = 2.25; SD = 1,130).

### Hypothesis testing

To determine the existence or not of differences according to gender and age (Hypotheses A and B), and to provide answers to the hypotheses posed, a Student’s t-test was performed. The resulting data can be observed in Tables 3 and 4.

**Table 3:** Student's t test and Cohen's d according to gender

		Gender	N	M	SD	Sig	T and Cohen's d
D.2	Item 17	Women	278	3.68	.721	.015	T = − 2.443; d = 1.12 differences in favor of women
		Man	153				
	Item 18	Women	278	3.49	.768	.004	T = − 2.883; d = − . 28 differences in favor of women
		Man	153				
	Ítem 20	Women	278	3.99	.647	.013	T = − 2.499; d = − . 25 differences in favor of women
		Man	153				
	Item 23	Women	278	2.52	1.129	.037	T = 2.097; d = .22 differences in favor of women
		Man	153				
D. 3	Item 25	Women	278	3.78	.784	.006	T = − 2.748; d = − . 29 differences in favor of women
		Man	153				
	Item 29	Women	278	3.90	.891	.003	T = − 3.005; d = − . 23 differences in favor of women
		Man	153				
D.5	Item 36	Women	278	4.16	1.152	.002	T = − 3.083; d = − . 31 differences in favor of women
		Man	153				
	Item 39	Women	278	2.82	1.149	.004	T = − 2.901; d = − . 30 differences in favor of women
		Man	153				
	Item 43	Women	278	2.47	.990	.017	T = 2.403; d = .24 differences in favor of women
		Man	153				
	Item 48	Women	278	3.38	1.107	.007	T = − 2.694; d = − . 28 differences in favor of women
		Man	153

**Table 4:** Student's t-test and Cohen's d according to the study degree

	item	Degree	N	M	SD	Sig	Student's t and Cohen's d
D.2	11	Infantile	361	3.87	.635	.000	T = − 4.260; d = − .22 in favor of Primary students
		Primary	70				
	12	Infantile	361	3.86	.643	.001	T = − 2.723; d = − . 12 in favor of Primary students
		Primary	70				
	1 5	Infantile	361	3.97	.636	.017	T = − 2.581; d = − . 32 in favor of Primary students
		Primary	70				
D.3	24	Infantile	361	4.00	.614	.000	T = − 4.256; d = − .45 in favor of Primary students
		Primary	70				
D.4	32	Infantile	361	3.73	.760	.016	T = − 4.372; d = − .52 in favor of Primary students
		Primary	70				
D.5	3. 4	Infantile	361	4.36	.917	.013	T = − 2.620; d = − .28 in favor of Primary students
		Primary	70				
	39	Infantile	361	3.80	1.246	.038	T = − 2.493; d = − .33 in favor of Primary students
		Primary	70				
	44	Infantile	361	2.91	1.049	.013	T = 1.020; d = − . 06 in favor of Infant students
		Primary	70				
	48	Infantile	361	3.21	1.075	.034	T = − 2.181; d = − .26 in favor of Primary students
		Primary	70				

#### **Hypothesis A**

 No statistically significant differences were found in the first, fourth, or sixth dimensions. With respect to dimension 2 (*Perceived utility of the PTM*) it was verified that women considered the Moodle platform as a tool that allowed them to learn in a simple manner and the class was relaxed [t = −2.443, *p* = 0.015, d = 1.12], and the interface was dynamic [t = 2.097, *p* = 0.037, d = 0.22], so that their learning was more independent, and thus they preferred to participate more in classes that used a blended format [t = −2.499, *p* = 0.013, d = −0.25]. At the same time, they preferred problems that they could work on without a defined scheme of contents through the PTM [t = −2.883, *p* = 0.004, d = −0.28].

As for the third dimension, (*PTM interface*), differences were found in favor of the female students, who mentioned that for them, the interface offered by the PTM was didactic [t = −2.748, *p* = 0.006, d = −0.29] and fast [t = −3.005, *p* = 0.003, d = −0.23]. Lastly, for dimension 5 (*Use of tools*), differences were also found in favor of the female students, who used the chat [t = −2.694, *p* = 0.007, d = −0.28], the forum [t = −3.083, *p* = 0.002, d = −0.31], and email [t = −2.901, *p* = 0.004, d = −0.30] features more often than the boys to communicate with their university peers and the professor. Also, they indicated that the wiki tools were used for personal academic activities [t = 2.403, *p* = 017, d = 0.24].

#### **Hypothesis B**

 In order to answer hypothesis B, the age variable was re-categorized, considering the following intervals: 18–19, 20–21, 22–23, 24–25, and 25. Next, an analysis of variance (ANOVA), based on the 6 dimensions already established in the EFA was performed. Note that dimensions 4 and 6 did not show any differences when using this variable.

Regarding dimension 1, differences were found in item 7 [F (4,426) = T = 2.810, *p* = 0.025, η^2^ = 0.026], indicating that students over 25 positively valued the ease with which they could identify where to consult the program of the course in the PTM [t (426) = 3.014, *p* = 0.027]. In the second dimension, similarly, differences were only found around item 22 [F (4,426) = T = 4.034, *p* = 0.003, η^2^ = . 037], where students aged 22–23 years-old said to be more in agreement than 18–19 years with the statement "I prefer to participate in blended-learning based courses " [t (426) = 3.30, *p* = 0.01]. The third dimension also showed a unique difference in item 26 regarding their opinion on the motivation of the PTM interface [F (4,426) = T = 2. 512, *p* = 0.0 41, η^2^ = . 023], with the students aged between 24–25 years-old the ones who manifested to be more in agreement with it [t (426) = 3.07, *p* = 0.02].

In the fifth dimension we found differences according to age in 5 of the items that comprised it.

Regarding the first of them, item 42 [F (4.426) = T = 3.108, *p*. = . 015, η^2^ = . 029)], students aged 18–19 years-old stated that they were more in agreement with the statement «I use podcasting for personal activities» [t (426) = 2.94, *p* = 0.034,] than those aged 24–25 years-old. For item 44 [F (4,426) = T = 4,777, *p* = 0.001, η^2^ = 0.044], as in the previous case, we observed that the differences were found among the youngest students (18–19 years-old) as compared to those who were older (24–25 years-old), as they mentioned being more satisfied with the use of wikis for personal activities [t (426) = 3.4 0, *p* = 0.007]. In the case of item 46 [F (4,426) = T = 2,557, *p* = 0.038, η^2^ = . 024] the youngest students [t (426) = 2.83, *p* = 0.046] expressed being more in agreement with the statement «I use blogs for academic activities» than those over 25 years old.

As in the two previous cases, in item 49 [F (4,426) = T = 2,810, *p* = . 025, η^2^ = . 019] it was the youngest students who reported using email to communicate with teachers [t (426) = 2,282, *p* = 0.047], unlike those from the 20–21 age group. Finally, it was in item 47 [F (4,426) = T = 5,073, *p* = 0.001, η^2^ = 0.047] where we found two differences between young people in the 18–19 group [t (426) = 4.29, *p* = 0.000] and those in the 20–21 age group [t (426) = 3.38, *p* = 0.008], as in both cases, the young people (18–21) agreed with the idea that they used the PTM chat to communicate with teachers.

#### **Hypothesis C**

 As shown in Table 5, differences were not found in dimensions 1 and 6 in our study sample, although differences were found in the rest of the dimensions. In dimension 2, statistically significant differences were found in the items «The activities performed through the PTM incite me to become involved in my own learning» [t = −4.260, *p* = 0.000, d = −0.22], «PTM promote research skills» [t = −2.723, *p* = 0.001, d = −0.12], and in « PTM facilitates active learning» [t = −2.723, *p* = 0.017, d = −0.32], with the evaluation by the Primary School Education students being higher that the Early Childhood students in all cases.

**Table 5:** Correlation between dimensions

		D.1	D.2	D.3	D.4	D.5	D.6
D. 1	R	1					
	P						
D. 2	R	. 502 (**)	1				
	P	. 000					
D. 3	R	. 495 (**)	. 567 (**)	1			
	P	. 000	. 000				
D. 4	R	. 390 (**)	. 526 (**)	. 401 (**)	1		
	P	. 000	. 000	. 000			
D. 5	R	. 334 (**)	. 376 (**)	. 272 (**)	. 291 (**)	1	
	P	. 000	. 000	. 000	. 000		
D. 6	R	. 388 (**)	. 435 (**)	. 454 (**)	. 348 (**)	. 205 (**)	1
	P	. 000	. 000	. 000	. 000	. 000	

**The correlation is significant at the level 01 (bilateral)

In dimension 3, differences were only found in the statement «It is easy to perform individual and collaborative activities through the PTM» [t = −4.256, *p* = 0.000, d = −0.45], with the Primary Education students being more in agreement as compared to the Early Childhood degree students.. This was also found for the item «The professors, through the PTM, suggest additional strategies so that we can better complete our activities» [t = −4.372, p = 0.016, d = −0.52], corresponding to dimension 4, in which the difference was in favor of the Primary School Education degree students.

Lastly, with respect to the fifth dimension, differences were found in the elements «I use the chat to communicate with my friends» [t = −2.620, *p* = 0.013, d = −0.28], «I use email to communicate with my University classmates» [t = −2.493, *p* = 0.038, d = −0.33], « I use the wikis for my personal activities» [t = 1.020, *p* = 0.013, d = −0.06], and «I use the chat to communicate with the professors» [t = −2.181, *p* = 0.034, d = −0.26], with the evaluation by the Primary School Education students being greater than those from the Early Childhood Education students.

Table 5. Correlation between dimensions.

## Discussion

At present, learning involves using the resources that the Internet makes available to students for this process, and teachers who promote the mobilization of basic skills in the students, so that their incorporation into the productive sector, once completed their higher education, can be as uncomplicated as possible. We agree with López-Belmonte et al. (2019), and García-Plana and Taberna-Torres (2021), that in the current situation, and in the next few years, teaching supported by PTM will become part of the higher education models, opening the doors to the university centers that are traditionally face-to-face, towards new student training scenarios, as well as the dissemination of the their scientific productions.

As far as online training mediated by the use of PTM is concerned, in this case Moodle, it is necessary to take into account the opinion that both students and teachers have of them, given that their beliefs and visions, together with their previous experience, will determine the overall attitude towards distance learning (Grande-de-Prado et al., 2021; Weidlicha & Bastiaensc, 2019).

In this sense, and focusing on university students, it has been shown that they have an attitude of positive indifference (de Morais et al. 2021; AUTHOR) towards this online teaching format; unlike results from Felpeto-Guerrero et al. (2015), Robinson et al. (2017) and Al-Fraihat et al. (2020), who indicated that students valued very highly and were mostly satisfied this form of learning.

In response to objective 1 (*To determine which are the most utilized tools of the PTM during their learning process*), the results indicated that students do not use all the available tools and that they opt for the use of blogs, email and Podscasting in their personal sphere, and email and chats in the academic sphere and with their teachers (Badía et al., 2019; de Morais et al., 2021), as compared to studies that indicate that the forums are the places where they find the most satisfaction for learning (Maz et al., 2012; AUTHOR).

Also, their assessment of both the usefulness of the platform itself and its interface was diverse (Objective 2. *Evaluate the PTM interface used by the university*). On the one hand, they considered that it allowed them to learn at any time (Wichadee, 2015) and, on the other, that the interface itself was valued in a moderately positive manner (Teo et al., 2019).

With respect to the third objective (*Assess the figure of the teacher against their learning process mediated by the PTM*), the university students using Moodle considered that his or her main role was to guide them on the subject, evaluating the work developed within it in a moderate manner (Chow et al., 2018), in contrast to the work by de Morais et al. (2021), which showed that the professors were not well-evaluated due to their scarce or low presence, and communication with the students (Mpungose, 2020). It would be necessary to verify whether or not this observation is altered at the end of the current confinement situation, or the current academic year in which the subjects were developed in this manner.

As for the evaluation of the Wi-Fi network (objective 4) offered to them by the University of Cordoba, the results obtained showed that it is not evaluated excessively high, which mirrors the difficulty experienced when trying to access it, even though it is an essential element that is needed to be able to work normally in online training, and which every Institution should ensure its optimal functioning (Aljawarneh, 2019; de Morais et al. 2021). However, even when their perception was not positive, they expressed that they connected to use the platform both from school, and from their homes.

Finally, for objective 5 (*The students of the Primary School Education Degree are more satisfied with the training through Moodle*), the results showed that in general, there were differences between both degrees in a number of issues. In the case of the items referring to: The activities that are managed through the PTM encourage me to become involved in my learning; The PTM promotes the ability to investigate; The PTM facilitates active learning; It is easy to carry out individual and collaborative activities through the PTM; The teachers, through the PTM, suggest additional strategies so that we can better complete our activities; I manage the chat to communicate with my friends; Handling e-mail to communicate with my university peers, and I manage the chat to communicate with teachers, the Primary School Education students were the ones who agreed with these statements, just as shown by the research carried out by AUTHOR. As we observe, the students believe that the Moodle PTM encourages them to be interested in improving their training process (Al-Fraihat et al., 2019; Binyamin et al., 2019). On the other hand, it is significant that the Early Childhood Education students only showed differences in their favor in the item which referred to the use of wikis as a tool they use for academic activities when, in general, it was not one of the tools valued as one of the most utilized, in agreement with the data of other researchers (Badia, 2019).

With regard to the hypotheses, in the case of Hypothesis A (*Women are more favorable to online training show*), this is accepted, as it has been shown that the female students were the ones who were more open to participate in online format courses (AUTHOR), and hypothesis B (*Younger students present *a *better assessment and use of the PTM*), it was proven that there were few differences based on this. However, the youngest students were the ones who used the tools mentioned above the most, as compared to the oldest learners.

Taking all this into account, we can conclude that the students, although they accept teaching mediated through the PTM, they are more accustomed to face-to-face teaching. For them, the platform is merely a repository of the information that teachers use in their classes and a place where they can search for it. This implies that at the present time in which all their learning has been converted to the online modality will require, now and for years to come, a greater degree of virtualization of content by teachers, as well as a greater involvement in the management of the platforms, given the unfriendly perception that the students have expressed.

## Conclusions

The main conclusions we have found, and which could be used for future studies once the health pandemic situation has ended, reveal that on the one hand, the structuration of the content in the platform is an important factor for its use, so that the Andalusian students satisfactorily considered the aspects related to the organization and accessibility of the different elements that comprised the different courses (objectives, methods, etc.), and without differences found in the opinion of the students, according to gender, age, or degree.

At the same time, although they felt indifferent about its use, their recognized its usefulness, especially with respect to the ease of being able to learn at any time, with most of the students preferring face-to-face classes as compared to a blended learning modality. Also, they asked for the professor to use the platform to monitor them, and an orientation session for the course in question, so that the platform is not used as a mere repository or for the submitting of classwork.

It should be highlighted that the potential for activities and tools it has should be further exploited, especially having in mind that the students only use those they know. The professors could propose practical activities or other types of work, making a greater use of the range of possibilities offered by the PTM, thus promoting ubiquitous learning, an aspect that the students considered to be very positive.

For the students, the interface of the tool they will work with is important, considering that the functionality for using the PTM does not invite its voluntary use, so that a more friendly and intuitive design should be provided.

Lastly, for the optimized use of these education tools, a good Internet connection is necessary. The results have brought to light that the Wi-Fi network offered by the university should be improved so that obstacles for its use are removed.

## Limitations

The main limitations found in the field of Social Sciences research is related to the sample size. However, in the case of the present study, it should be taken as a starting point, which could be used to verify the results in other degrees and universities.

Moreover, the instrument used was designed so that it can be administered to the teaching staff from not only this university, but to others as well. Its use could allow us to draw a current map of the actual use of these platforms in order to develop training programs which respond to their concerns about it and, with it, achieve the functionality for which it was created.

## Abbreviations

ANOVA: Analysis of variance
CRUE: Conference of Rectors of Spanish Universities
EFA: Exploratory factor analysis
EHEA: European Higher Education Area
M: Media
N: Sample
PTM: Teletraining platforms
R: Performed the Pearson
SD: Standard Deviation
ICT: Information and Communication Technologies
UNED: National Distance Education University
UOC: Open University of Catalonia
